# Critical Review of Food Colloidal Delivery System for Bioactive Compounds: Physical Characterization and Application

**DOI:** 10.3390/foods13162596

**Published:** 2024-08-19

**Authors:** Bijie Wang, Jiayi LvYe, Shaoming Yang, Ying Shi, Qihe Chen

**Affiliations:** 1 Department of Food Science and Nutrition, Zhejiang University, Hangzhou 310058, China; 22213056@zju.edu.cn (B.W.); ouoyeh@163.com (J.L.); shiying0520@zju.edu.cn (Y.S.); 2 Zhejiang Longquan ZhengDa Biotech Co., Ltd., Lishui 323000, China; 13362093177@163.com; 3 Innovation Center of Yangtze River Delta, Zhejiang University, Jiashan 310000, China

**Keywords:** food colloid, delivery system, physical characteristic, application, structure

## Abstract

Bioactive compounds (BACs) have attracted much attention due to their potential health benefits. However, such substances have problems such as difficulty dissolving in water, poor stability, and low intestinal absorption, leading to serious limitations in practical applications. Nowadays, food colloidal delivery carriers have become a highly promising solution due to their safety, controllability, and efficiency. The use of natural macromolecules to construct delivery carriers can not only regulate the solubility, stability, and intestinal absorption of BACs but also effectively enhance the nutritional added value of functional foods, improve sensory properties, and extend shelf life. Moreover, smart-responsive colloidal delivery carriers can control the release characteristics of BACs, thus improving their absorption rate in the human body. This review describes the characteristics of several typical food colloid delivery carriers, focuses on their physical properties from static structure to dynamic release, summarizes their applications in delivery systems, and provides an outlook on the future development of food colloid delivery carriers. The different compositions and structures of food colloids tend to affect their stability and release behaviors, and the different surface properties and rheological characteristics of the carriers predestine their different application scenarios. The control of in vivo release properties and the effect on food media should be emphasized in the future exploration of safer and more controllable carrier systems.

## 1. Introduction

With the continuous improvement in people’s living standards, humans’ demand for food is not only limited to solving the needs of hunger and taste, but its health and functional properties are also gradually emphasized [1]. A number of bioactive compounds (BACs), such as natural plant extracts, fatty acids, vitamins, proteins, bioactive peptides, active polysaccharides, and oligosaccharides, have been receiving attention for their potential health benefits in reducing the risk of diseases such as diabetes, cardiovascular disease, and obesity [2]. However, if such BACs are directly dispersed in the food system, not only do they need to be considered for their compatibility with the food system and whether they will chemically interact with other ingredients in the food [3], some highly sensitive BACs such as carotenoids and vitamins are also highly susceptible to inactivation during processing and storage [4]. Additionally, BACs with undesirable flavors such as tannins, capsaicin, etc., as well as some large insoluble particles can also have a negative impact on the flavor of food products [5]. Colloidal delivery systems offer a promising solution to this problem, as BACs can maintain their insoluble or bad-flavored properties in colloidal form, withstand the effects of undesirable external factors (e.g., light, heat, oxygen, pH, etc.), and at the same time reduce the physical and chemical reactions with other substances in the surrounding medium [2,6,7]. In addition, BACs loaded in a designed colloidal system are resistant to the digestive environment and have improved permeability through the intestinal epithelium, resulting in better absorption and utilization by the body [8]. In the pharmaceutical field, colloidal delivery systems have provided alternative formulation methods for drug candidates for decades, from conceptualization to the identification of novel carrier materials, to passive and active targeting, and more recently to biointeractive systems [9]. On this basis, the application of colloidal carriers to the food industry is an extremely effective means of improving food quality and safety [1]. In this regard, the development of safe, edible, stable, and controllable food colloidal systems as delivery vehicles for such BACs is of broad research interest.

The colloidal delivery system encompasses a broad range of dispersion systems ranging from submicron emulsions to colloidal particles to liposomes and micelles [1]. Micelles can be below 100 nm in size and other dispersions can be around hundreds of nanometers in size [1]. Food-sourced polymers [10], including whey proteins [11], casein [12], soy proteins [13], gelatin [14], corn alkyd proteins [15], starch [16], and cellulose [17], are suitable raw materials for the creation of food-grade colloidal delivery vehicles (Table 1). These polymers have been engineered into different structures of colloidal delivery carriers according to their properties and show unique functional properties in the final product system. For example, by constructing micellar-type carriers that are externally hydrophilic and internally hydrophobic, hydrophobic BACs can be encapsulated into their hydrophobic cores through hydrophobic interactions, which greatly improve their water solubility and stability [18]. Various nano-/microencapsulated delivery systems can protect BACs from degradation in the gastrointestinal tract, thereby improving their absorption in the small intestine; complex coacervates can be effective in prolonging the retention time of flavor compounds to improve the organoleptic quality of food or masking the undesirable taste of functional factors [19]. Self-assembled nanoparticles show great advantages in mucus permeability, controlled-release properties, etc. [20,21].

We searched PubMed, Web of Science, and Scopus databases to summarize the major studies related to food colloidal delivery systems in the last 5 years. There are many studies and reviews on food colloidal delivery carriers, demonstrating the advantages of different colloidal carriers. However, in this paper, we would like to start with a few of the most common colloidal delivery vehicles available today and to describe and compare the characteristics, current development, and advantages and disadvantages of the different colloids. On this basis, the physical properties of these colloidal systems, from static to dynamic, are summarized, with special reference to the possible impact on the physical properties of the product matrix during practical application. Finally, possible application scenarios and future perspectives of colloidal delivery carriers are proposed with respect to the problems and challenges encountered in the application of most BACs.

## 2. Typical Structure of Food Colloid Delivery Carriers

**Figure 1:**
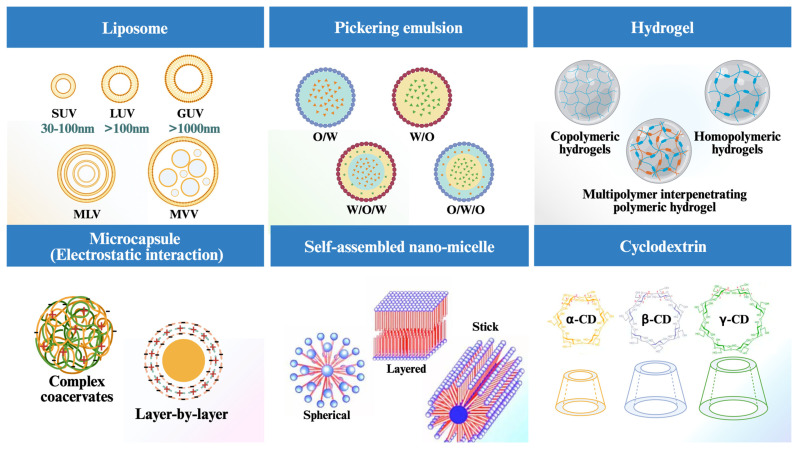
Representative food colloidal delivery carriers. (SUV: small unilamellar vesicle; LUV: large unilamellar vesicle; GUV: giant unilamellar vesicle; MLV: multilamellar vesicle; MVV: multivesicular vesicle; O/W: oil-in-water; W/O: water-in-oil; W/O/W: water-in-oil-in-water; O/W/O: oil-in-water-in-oil; cD: cyclodextrin).

BACs delivery carriers play an important role in the food industry. Natural food macromolecules such as polysaccharides, proteins, and lipids often form food colloidal delivery materials through self-assembly behavior, hydrophobic interactions, internal molecular bonding, and hydrogen bonding [38]. Delivery carriers can be prepared in a variety of structures such as liposomes, composite cohesive microcapsules, Pickering emulsions, hydrogels, nanomicelles, β-cyclodextrins, etc. (Figure 1) [39]. Delivery carriers based on different structures are discussed in detail next.

### 2.1. Liposome

Liposome is a colloidal encapsulation system; phospholipid molecules such as soy soft phospholipids and milk phospholipids are the main components that make up the liposome, and sometimes, sterols, chitosan, etc. are added in order to stabilize the vesicle membrane [40,41,42]. Based on the number and structure of lipid bilayers, liposomes are categorized into three types: unilamellar vesicles (ULV), multilamellar vesicles (MLV), and multivesicular vesicles (MVV) (Figure 1) [43]. Liposome technology can be used to improve the solubility and bioavailability of most BACs and to achieve controlled release at precise sites [44]. Therefore, this technology plays a crucial role in functional food, nutritional, and pharmaceutical applications [45]. These BACs include shrimp oil [22], carotenoids [23], phenolics [6], sterols [41], bioactive peptides [46], and vitamins among others (Table 1). Generally, fat-soluble BACs are encapsulated on the phospholipid bilayer of liposomes, whereas water-soluble BACs are encapsulated in the aqueous phase within liposomes [7]. Liposomes have a very similar structure to the cell membrane and can cross it efficiently, which gives them an efficient delivery role [47]. Liposomes are cost-effective compared to other colloidal delivery systems and are one of the most common commercially available lipid carriers; however, they are physicochemically unstable due to the fact that lipids present in their structure can be naturally degraded by oxidation or hydrolysis, and even form agglomerates [48]. In addition, phospholipids are vulnerable in the gastrointestinal tract (GIT), especially in the intestinal region. The in vivo stability of liposomes can be enhanced by the addition of hydrogenated lipids, fluorinated lipids, and cholesterol [7,49]. Today, innovative preparation techniques are also driving improvements in liposome stability [50,51,52]. For example, Shashidhar et al. used the supercritical anti-solvent method to mimic the traditional method (thin-layer dispersion method) to achieve the encapsulation and release of adenosine, cordycepin, and polysaccharides in *C. sinensis* CS1197. The process was optimized using ScCO_2_ at a depressurization rate of 25 bar min^−1^ to obtain liposome structures with smaller size and more stable physicochemical properties [53]; the replacement of organic solvents in the reverse-phase evaporation method by ScCO_2_ also improves the encapsulation efficiency of BACs [54]. In addition, green technologies such as microfluidization and ultrasonication provide better control of the physical properties of liposomes, which is advantageous in the production of high-end liposomal products and efficient process scale-ups [55,56].

### 2.2. Pickering Emulsion

Pickering emulsions (PEs) are emulsion systems formed by the aggregation of colloidal particles instead of conventional surfactants at the oil–water interface [57]. Commonly used food-grade colloids include protein/polysaccharide complexes, starch/fat crystals, flavonoids, plant waxes, and a variety of colloidal structures including microgels, food-based nanoparticles, protofibers, etc. [26,27,58,59,60,61] The tight arrangement at the oil–water interface gives Pickering emulsions excellent stability, as well as multi-class structure, high specific surface area and selective permeation [62]. The basic PEs can be categorized into water-in-oil (W/O) systems and oil-in-water (O/W) systems, and the multiple PEs (W/O/W and O/W/O) built on this basis belong to more complex systems (Figure 1) [63]. They are important for encapsulating and releasing water-soluble BACs and fat-soluble BACs, respectively. Nowadays, modified starch granules are considered as a suitable substance to stabilize multiple PEs due to their microstructure and physicochemical properties [64]. For example, hydrophilic colorants (carmine) encapsulated in starch modified with octenylsuccinic anhydride as well as shea oil encapsulated in modified quinoa starch show high stability [65,66].Multiple PEs also have the ability to capture and protect nutrients and facilitate their controlled breakdown for delivery through the digestive system, making multiple PEs attractive for food applications. There is a proliferation of novel alternate delivery materials constructed using multiple PEs as templates today. For example, Boostani, et al. fabricated medium- and high-internal-phase W1/O/W2 multiple PEs using physically modified hordein nanoparticles at a dispersed-phase volume fraction (Φ) of 0.5 with an overrun value of ~40%, creating highly promising candidates for multi-structured colloidal systems [67]. Multiple PEs can also be used as facile templates for the preparation of multi-hollow nanocomposite microspheres with better stability and barrier and release properties compared to emulsions [68,69]. However, there are some limitations of multiple PEs that need to be improved, such as high cost, easy decomposition, and susceptibility to osmotic pressure [28]. Moreover, Pickering high-internal-phase emulsion (HIPE), which is regarded as a concentrated version of PE, can accommodate a large number of BACs and is a good candidate for the controlled transport of BACs, and the colloid exhibits a G′ greater than G″ at low strain and can also be used for food-grade 3D printing. However, due to its high-dispersed-phase content, it is usually technically challenging to stabilize HIPEs with large amounts of low-molecular-weight surfactants [70]. In addition, PEs are difficult to equilibrate during the preparation process; it is difficult to form the final desired droplet size, which limits their industrial production, and their biocompatibility is yet to be improved, limitations that need to be attended to in order to enhance their application potential [71].

### 2.3. Hydrogel

**Figure 2:**
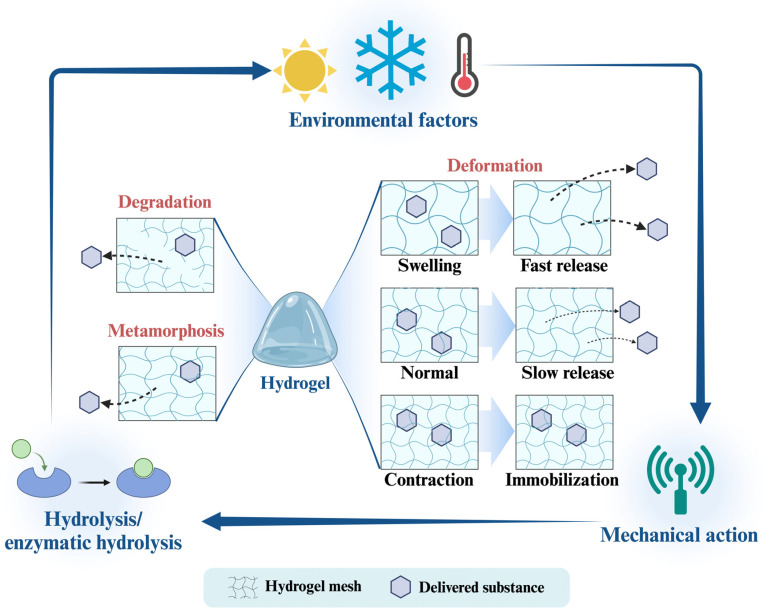
Hydrogel delivery mechanism.

Hydrogels consist of a porous, three-dimensional network of polymer molecules that contain large amounts of water, making them relatively soft, flexible, and viscoelastic materials [72]. Hydrogels act like extracellular interstitials in living tissues and therefore are generally well biocompatible [73]. Some proteins/polysaccharides (gelatin, casein, zeinolysin, sodium alginate, pectin, etc.) derived from animals, plants, or microorganisms can be used to prepare hydrogels with good biocompatibility, with the disadvantage of poor mechanical properties [74]. In this regard, hydrogels prepared from some synthetic polymers including polylactic acid, polyhydroxyacetic acid, polyoxymethylene, and polyethylene glycol, on the other hand, show better physical stability but poor biodegradability [75]. Different particles can be utilized in combination to obtain advanced hydrogel complexes with different mechanical and functional properties by design (homopolymeric hydrogels, copolymeric hydrogels, and multipolymer interpenetrating polymeric hydrogel) (Figure 1) [76]. These hydrogels also have some unique properties such as stimulated response and bioadhesion [77]. When exposed to specific environmental conditions (e.g., pH, ionic strength, temperature, enzymes, etc.), hydrogels can be engineered to swell or disintegrate, which is effective for targeted delivery applications (Figure 2) [78]. For example, Song et al. prepared a pH-responsive semi-IPN hydrogel consisting of peach gum polysaccharide (PEP) and *Auricularia polytricha* β-glucans (APP) by inverse emulsion cross-linking method [30]. Due to the stable and dense structure of the PGP-APP semi-interpenetrating network hydrogel, there was a significant positive effect on bacterial viability in a gastrointestinal transformation model (*p* < 0.05), and this work also illustrates the potential of PEP-based hydrogels for application as intestinal-targeted delivery carriers. Hu et al. developed novel polyelectrolyte complex hydrogel based on self-assembly of Salecan and *N*,*N*,*N*,*N*-trimethyl chitosan, and the hydrogel based on electrostatic interactions provides a suitable carrier for intestinal-targeted delivery of green tea polyphenols [31]. In addition, the surface of hydrogels can be made into bioadhesives to increase their retention time within the GIT [77], and the use of oxidative modifications can improve the charge density and controlled-release properties of electrically neutral polysaccharide-based hydrogels [79]. The combination of different technologies can be used to optimize the performance of delivery vehicles. For example, the combination of gel particles and complex emulsions can be used to construct a synergistic delivery system of multiple functional factors with embedded emulsions [80], and the combined structure of gel particles and LbL layers can be used to regulate the release more effectively [81]. It is worth mentioning that some of the current hydrogel carriers still suffer from low loading rates, unstable release rates, and difficulties in ensuring the activity of BACs [82]. In addition, hydrogels tend to adsorb a large amount of water and swell, so attention needs to be paid to the interaction between the gel and other components of the food carrier to ensure that the introduction into food products will not adversely affect the quality of the products.

### 2.4. Self-Assembled Microcapsules Based on Electrostatic Interaction

Complex coacervates are special structures formed by electrostatically charged polymer particles due to the liquid–liquid phase separation phenomenon (LLPS), a process accompanied by an entropy increase caused by the release of counterions (Figure 1) [83]. Complex coacervates are generally produced by positively charged proteins and negatively charged polysaccharides; e.g., forming a complex cohesive layer of Arabic gum and β-lactoglobulin was one of the early reports [84]. Several studies have shown that the viscoelastic property of the complex coacervates is an effective indicator for characterizing the strength of electrostatic interactions between polymer particles in the cohesive layer [85,86,87]. The main advantages of this technology are its high encapsulation rate (>99%) and excellent sustained release properties based on mechanical stress, temperature, etc. [83]. Although this approach has potential applications in the field of BACs delivery, it has some limitations. First, the stability of the composite coalescing microgels may not be high enough, which means that they may decompose or change during storage or use, thus affecting the delivery effect. Second, due to the multiple factors involved in the composite coalescence process (pH of the system, polymer concentration, polymer mixing ratio, ionic strength, and heat treatment), its controllability is relatively low, making it difficult to precisely control its structure and function [88,89,90]. The morphology and size of complex coacervates are related to their processing, and usually, low homogenization rates form mononuclear microcapsules, while high homogenization rates form multinuclear microcapsules [91]. Complex coacervates are mainly used to encapsulate hydrophobic compounds and have some limitations for encapsulating hydrophilic substances. It has been indicated that if a double emulsion step is added at the beginning of the preparation process, it can lead to the encapsulation of hydrophilic compounds [92].

Moreover, multilayer microcapsules formed by layer-by-layer (LbL) technology, which relies on electrostatic interactions, are also commonly used to encapsulate and control the release of functional factors (Figure 1) [93]. Under the right conditions, cationic polyelectrolytes can over-adsorb on the negatively charged surface of a solid carrier, resulting in a surface charge reversal. This process is self-limiting and highly reproducible, ultimately allowing for the continuous fabrication of layered structures. Proteins and polysaccharides are commonly used as preparation materials. Soybean isolate protein, modified starch, and chitosan have been used to prepare triple-layered microcapsules via the LbL assembly technique, which have lower encapsulation efficiencies compared to single-layered or bilayered microcapsules, but they exhibit programmed- and controlled-release behavior [94]. Notably, LBL assembly is driven by, but not limited to, electrostatic interactions but also by other forces including hydrogen bonding and biospecificity, thus expanding the materials used for thin-film structures, the deposition conditions, and the properties of the films [95]. However, LbL assembly techniques have some drawbacks, such as the difficulty of controlling the preparation process and the dissociation of the structure due to salt sensitivity, which limit their application in food products. In response to this, additional covalent cross-linking can usually be introduced between the layers to stabilize the structure of the LbL layers.

### 2.5. Self-Assembled Nanomicelle

Micelles are nanoscale aggregates formed by self-assembly of amphiphilic polymers in aqueous solution [96]. Low concentrations of amphiphilic polymers exist as monomers. As the concentration approaches the critical micelle concentration, the amphiphilic polymer aggregates to form micro-/nanoclusters with regular morphology (tubular, spherical, etc.) (Figure 1) [97]. The assembly of these molecules is mediated by the orientation of these groups in a suitable environment. Specifically, in polar solvents, the hydrophilic portion of the molecule faces outward to maximize contact with the polar solvent, and the hydrophobic portion aggregates in the core to accommodate the water-insoluble drug, a structure known as normal nanomicelles. Conversely, in nonpolar solvents, the structure is called reverse nanomicelles [98]. Currently in the food industry, amphiphilic polymers such as polysaccharides (e.g., chitosan, dextran, starch, maltodextrin), casein, and surfactants (e.g., SDS, Tween) modified by hydrophobic groups are commonly used for the preparation of self-assembled nanomicelles (Table 1) [99]. Nowadays, it has also been shown that lipid hydrolysates and bile salts can jointly contribute to the formation of mixed micelles of bile salts during digestion [100]. Bile salts and bile acids have been extensively investigated for the preparation of amphiphilic polymers; e.g., deoxycholic acid–dextran polymers were synthesized by dipolar 1,3-cycloaddition reaction and self-assembled to form micelles, and an increase in the water solubility, a diminution of the bursting effect, and a slow release of curcumin could be achieved by encapsulating curcumin in the micelles using a dialysis method [101]. Compared to other colloidal delivery systems such as emulsions and liposomes, micellar systems are characterized by their small size, homogeneity, and thermodynamic stability, which gives them a unique advantage as delivery systems for BACs [102]. The particle size of polymeric micelles, typically between 10 and 200 nm, allows for complete absorption via endocytosis, thus enhancing the bioavailability of encapsulated BACs. For example, re-assembled casein-based micelle-loaded vitamin D bioavailability was increased 4-fold [103], and hyaluronic acid–octadecanoic acid-based micelle-loaded curcumin bioavailability was increased 1,7-fold [104]. In addition, due to the inherent self-assembly properties of micelles, micellar systems are easier to prepare than other colloidal systems and are widely used as encapsulants, protein solubilizers, enzyme stabilizers, etc. in the food industry [99]. However, micellar systems are sensitive to various conditions such as temperature, ionic strength, and enzymes, so in-depth studies on maintaining the stability of micelles under specific conditions are crucial for their application.

### 2.6. Cyclodextrin

Cyclodextrins (CDs) are cyclic oligosaccharides composed of glucopyranose units, which are “hollow, decapitated, conical cylinders” of molecules consisting of more than six glucoses linked by α-1,4-glycosidic bonds. The natural form of cyclodextrins consists of 6, 7, or 8 glucopyranose units, named α-, β-, and γ-cyclodextrins in that order, with a cavity volume of 0.174, 0.262, and 0.427 nm^3^ (Figure 1) [105,106]. They are authorized as food additives, and in general, β-CD and its derivatives are the most widely used [107]. Inorganic and organic salts, as well as neutral molecules, can form complexes with CD, but they are more commonly used to bind insoluble BACs, producing so-called “inclusion complexes” [108]. In general, cyclodextrins interact with biologically active compounds to enhance their solubility or stability, such as the complexes formed by natural cyclodextrins with antioxidants, vitamins, saponins, fatty acids, or carotenoids [109]; they can also control the release of volatile molecules and even eliminate undesirable odors [110]. In addition, in terms of food packaging materials, cyclodextrin also has a wide range of potential applications. Taking oregano essential oil–β cyclodextrin inclusion compound as an example, active food packaging materials are prepared by electrostatic spinning technology, which can effectively extend the shelf life of fruits and vegetables [111]. Moreover, CEO-β cyclodextrin inclusion complex, for example, combined with nano-liposome technology, can effectively improve the shelf life of meat products and develop new antimicrobial packaging materials by inlaying casein as a target receptor for bacteria [112]. However, despite the many advantages of cyclodextrin encapsulation technology, the chemical composition of BACs remains essentially unchanged before and after cyclodextrin encapsulation in practical applications, but the ratio of each component changes to varying degrees. Moreover, the dissolution rate of BCAs in the inclusion complexes is difficult to guarantee, thus limiting their effectiveness in some specific applications [113].

## 3. Physical Properties of Food Colloid Delivery Carriers

It is necessary to emphasize the physical properties of food colloid delivery systems. The particle structure, surface properties, and microscopic/macroscopic mechanical properties of food colloids are typically important factors affecting their loading and delivery efficiency and their stability in the product system [89]. The physical properties of food colloid delivery systems with different structures have been summarized in Table 2.

### 3.1. Size and Structure

The size and structure of food colloid delivery systems depend on the composition, assembly conditions and preparation techniques used to prepare them. Typically, the size of these colloids varies from a few nanometers to a few micrometers and can be characterized using physical parameters such as particle size distribution (PSD) or mean particle size (d) and polydispersity index (σ). Knowing the critical size of different colloids is important for studying their structure [114]. It is worth mentioning that smaller sizes impart many beneficial biological properties to colloids; for example, the endocytosis process of polymer micelles generally increases with decreasing particle size [115]. However, smaller particles typically have a higher free surface energy, which may lead to reduced aggregation and stability of the colloidal system [116].

**Figure 3:**
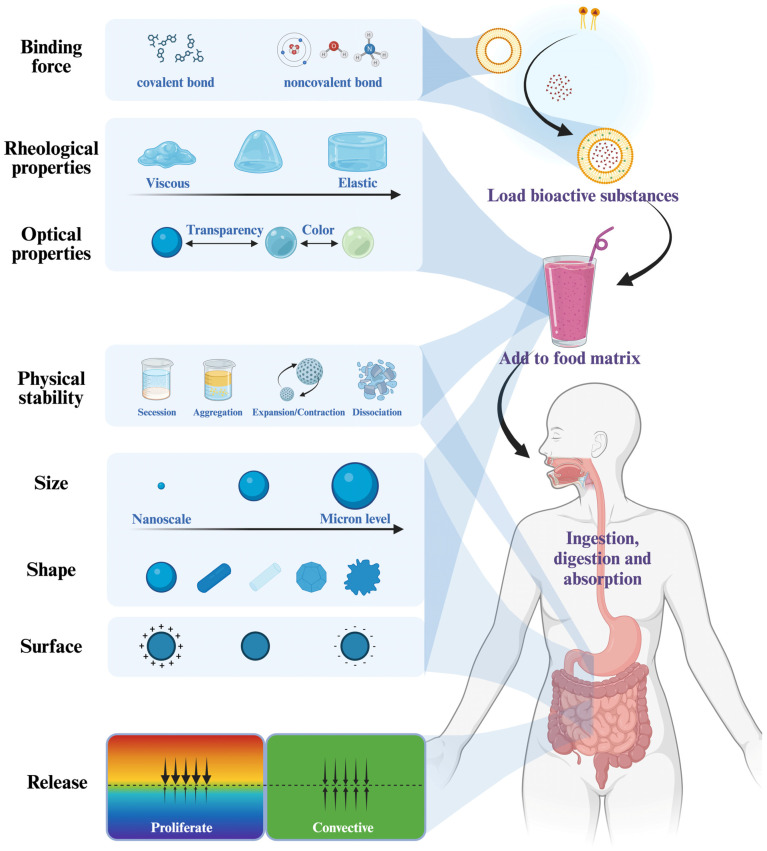
Physical properties of food colloidal delivery carriers.

In addition, shape is the macroscopic structure of the colloid and is the most fundamental property we can assign to it to change its interaction behavior. This function is even more pronounced in the case of colloidal self-assembly, which regulates particle–particle, substrate, and interface interactions [117]. Colloidal particles are mostly spherical, rod-shaped, or clustered (Figure 3), and usually colloidal dispersion systems with non-spherical particles have more complex rheology, diffusion in the gut and release patterns than colloidal dispersions with spherical particles of similar concentration. It is worth mentioning that the porosity of colloidal particles greatly affects the accessibility of BACs encapsulated in them. Altering the structure of colloids through innovative preparation techniques and parameters is a direct means of tuning colloid encapsulation efficiency, carrying capacity, permeability, integrity, environmental responsiveness, and digestibility.

### 3.2. Charged Characteristics

The charging properties of food colloids used as delivery vehicles usually depend on the charging properties of the component polymers in them [118]. Therefore, by varying one or more components carrying different charges and their concentrations is a common method used to control the overall charged properties of colloidal particles [119,120]. The charged properties of colloidal particles are also affected by factors such as pH, ionic composition, and dielectric constant of the surrounding environmental medium [121]. The charged condition of the colloid has an important impact on the binding interaction between it and the BACs and its degree of disassociation in the system [118]. In specific application scenarios, this charged property equally influences the form of colloids in food systems and their interaction with the surface of the human digestive system. Specifically, if colloidal particles carry an opposite charge to that of an ion of a food component in the food, then they may form electrostatic complexes and aggregate and precipitate. In the digestive tract, the situation is more complex. For example, colloidal particles carrying cations will readily bind to anionic surfaces on the tongue, thereby causing perceptible astringency [122]. Moreover, gastrointestinal fluids have relatively high ionic strengths (typically 100–200 mM) and contain a range of mono- and polyvalent ions (e.g., sodium, potassium, calcium, chloride, and bicarbonate). The presence of these mineral ions may alter the electrostatic interactions of colloidal particles with the inner surface of the gastrointestinal tract, thereby affecting mucus adhesion and mucus layer transport of colloidal particles [123].

### 3.3. Binding Characteristics

Both between the polymer molecules that make up the colloidal delivery vehicle and between the BACs and the colloidal particles, substances tend to bind through interaction forces. Intermolecular interactions typically include covalent forces and non-covalent interactions (Figure 3) [124]. Among them, covalent interactions are highly specific, establishing permanent binding and irreversible interactions between proteins and polysaccharides; non-covalent bonds, such as van der Waals forces, hydrogen bonding, hydrophobic interactions, electrostatic interactions, etc., are nonspecific and act mainly in the form of attraction and repulsion [125]. For food colloidal delivery vehicles, the nature of the groups of the polymers in them determines the form of assembly of the colloidal particles. Some polymer particles are able to form ordered structures (molecules, colloids, micelles, etc.) spontaneously by using interaction forces, but some polymers are used to enhance binding and molding by using special processes (e.g., spray drying, solvent desorption, microfluidic methods, emulsion-templating methods, emulsion-templating, etc.) to enhance bonding and molding [10]. The loading of BACs into colloidal structures is also binding-force-directed, and there may be multiple binding sites within a colloidal structure. Understanding the number of binding sites and their affinity for ligand molecules is important for developing effective delivery systems that encapsulate specific types of BACs. A complex mathematical analysis of ligand–receptor binding in this colloidal delivery system has been performed, and the binding constants of several polymers that can be used to prepare colloidal carriers for BACs have been reported [123]. For example, the binding constant data of various polyphenol molecules to certain proteins are in the range of 500–350.000 M^−1^ [126], whereas the binding constant data of various bitter compounds to cyclodextrins are in the range of 0.01–10.000 M^−1^ [127]. In addition, there are a number of factors affecting the interactions between polymers, colloids, and BACs, including internal (e.g., pH, ionic strength, conformation, charge density, and concentration) and external (e.g., pH, ionic strength, temperature, nature of the biopolymer, and mass ratio) factors. Among other things, changes in pH may alter the sign and magnitude of charges on BACs and colloidal particles and increases in ionic strength may weaken any electrostatic interactions due to electrostatic shielding effects. As a result, some BACs stored within the aqueous phase may be released upon changes in ambient pH or ionic concentration [128,129]. Heating often breaks hydrogen bonds, so BACs loaded within colloidal particles based on hydrogen bonds may be released due to temperature changes [129]. Overall, the form and degree of action of the bonding forces are an important basis for further studies of the structure, drug loading and delivery characteristics, stability, and mechanical properties of food colloidal delivery systems.

### 3.4. Physical Stability

The stability of a colloidal particle is usually defined as its ability to maintain its composition and structure under certain media conditions (e.g., pH, ionic strength, and temperature) [130]. Typically, colloidal particles are supposed to maintain their integrity under one condition but break down under another for the process of drug loading and delivery of biologically active substances [131]. The main mechanisms of destabilization of biopolymer particles include gravitational separation (emulsification or precipitation), aggregation (flocculation or polymerization), volume change (expansion or contraction), and dissociation (erosion or decomposition) (Figure 3) [1]. These instabilities are sometimes due to physical factors (e.g., food processing heat treatment) and sometimes due to chemical factors (e.g., pH and ionic concentration) [131]. For the stability of colloidal particles, there are two main requirements: the first is that the colloids loaded with BACs should remain stable in any delivery system, that is, the food carrier throughout the shelf life, and the second is that the colloids loaded with BACs need to maintain their stability in the human digestive system before they reach their targeting destination. Some studies have shown that food-grade particle-stabilized PEs have more stable properties. For example, PEs stabilized by OSA-modified quinoa starch granules have very large droplet diameters (50–100 microns) but remain stable after more than two years of storage, and other suitable food-grade particles include soy protein-based gel particles, cellulose-based particles, and more [132,133]. If necessary, the colloidal structure can also be modified for the application; e.g., surface modification of liposome particles with sugars and their derivatives, polymers, or proteins can improve the stability of the liposome particles and their interaction with human tissues [134]. Among them, sugars and their derivatives have been studied more for their physical and chemical stability, for example, chitosan has a great potential for application in liposome surface modification due to its unique adhesion ability in the intestinal environment and controlled release [135]; hard xanthan gum promotes the static stability of liposomes by resisting the Brownian motion of lipid droplets; soft guar gum prevents contact between liposome vesicles [136]. The synergistic effect of all three has been shown to be effective in improving the stability of liposomal systems during long-term storage [137]. Moreover, sodium alginate-coated liposomes can improve their stability in the gastric environment [138]. It is worth noting that the safety of the modification methods as well as the effect on the appearance, texture, and flavor properties of the food system need to be considered simultaneously.

### 3.5. Release Behavior

The main forms of release from colloidal delivery systems include simple diffusion, fragmentation, swelling, or erosion, corresponding to the mechanism of colloidal particle instability, so that the behavior is usually dependent on the components of the biopolymer, the intermolecular interactions, and the changing physicochemical conditions in the environment. In this case, BACs diffuse directly into the surrounding medium due to differences in their solubility in the carrier system and in the environment, and the colloidal particle matrix remains intact. Swelling/shrinkage is usually caused by deformation of the particles due to solvent uptake/excretion by the colloidal particles, which causes the release of BACs from the inside, and in particular, in hydrogel systems, swelling or shrinkage of the particles causes a change in the size of the gel lattice, which leads to the leakage of active substances [73]. Erosion is the dissociation of physical bonds or hydrolysis of covalent bonds due to physical, chemical, or enzymatic degradation; fragmentation refers mostly to the release of BACs caused by physical fragmentation (by applying pressure, shear, etc.), and they usually lead to the disruption of the structure of the colloidal delivery vehicle [10]. There is a close relationship between the location of the BACs’ encapsulation and their release behavior; e.g., in the case of a liposomal structure, the BACs in the internal aqueous phase are rapidly released upon liposome rupture, whereas drugs on bilayers, which can remain encapsulated on fragments after liposome rupture, achieve slow release as the lipid fragments degrade [139].

In general, molecular motion is the result of diffusion or convection, with diffusion-induced molecular motion being a random motion caused by the thermal energy of the system, while convection-induced molecular motion is usually the result of fluid flow (Figure 3) [123]. Determining the form of molecular motion of BACs is of prime importance for studying their release characteristics and designing relevant delivery vehicles. Studies have been conducted to model the release mechanism of colloidal delivery systems of different shapes and structures based on mathematical theory [140,141]. The parameters required for these models include the size distribution of the initial particles, the concentration of the active component in the carrier and the surrounding medium (equilibrium partition coefficients), and the rate of transport of the active component through the system (advection–diffusion coefficients). The parameters required for these models include the size distribution of the initial particles, the concentration of the active component in the carrier and the surrounding medium (equilibrium partition coefficients), and the rate of transport of the active component through the system (advection–diffusion coefficients) [123]. However, most of the research bases of these simulations are from drug delivery systems, and research in food systems is still lacking, and further refinement of these models in the future will be of extraordinary significance for the study of the design, manufacture, and application of food systems with colloidal particles with specific release properties.

### 3.6. Effects on the Physical Properties of Food Substrates

#### 3.6.1. Rheological Properties

The incorporation of colloidal particles may positively or negatively affect the rheological or textural properties of the food matrix. In general, the rheological properties of colloidal suspensions in fluidized food systems depend on the concentration, shape, and interaction of the colloidal particles. The modulus of the system (storage modulus and loss modulus) increases with increasing colloid concentration until the colloidal particles are tightly packed together, i.e., a critical packing parameter (φ_c_) is reached [142]. If the concentration of colloidal particles exceeds φ_c_, the system will exhibit solid-like properties with a yield stress and an elastic modulus that exceeds its viscous modulus. In terms of shape, non-spherical colloidal particles have additional energy consumption compared to spherical ones, and therefore, the viscosity of the food system increases [143]. The rheology of colloidal dispersion also highly depends on the interaction between colloidal particles. If the particles are attracted to each other, then the suspension will be more dense and even more gel-like [144]. Interestingly, for the future development of food products with health benefits, colloids loaded with BACs might also be used as modifiers of food texture to provide desirable rheological properties to the product, such as thickening, etc., whereas the issue of an appropriate dosage might need to be considered [1]. For example, Xu et al. investigated the effect of flaxseed gum (FG) on the rheological properties and physicochemical stability of whey isolate protein (WPI)-stabilized β-carotene emulsions (β-carotene content of 0.02%) at pH 3.0. It was shown that the addition of 0.1 wt% FG significantly improved the physical stability of the emulsion, at which time the solid-fat balance and fluidity index of the system were significantly reduced, and the elasticity index was increased up to 3.7 times [145]. In addition, multiple Pes can be introduced as new formulations for the production of alternative foods. In a recent study, by adding whey, rice, and pumpkin seed proteins as fat substitutes, these fat substitutes had different physicochemical, structural, and mechanical properties, and these low-fat food formulations were suggested for use in healthy and nutritious diets [146].

#### 3.6.2. Optical Characterization

The polymer particles that make up the colloidal delivery vehicle interact with light waves, and their introduction into food may affect the overall opacity and color of the product and thus the appearance of this food presentation. The overall effect of colloidal particles on the optical properties of food often depends on their concentration, size, and refractive index. Small particles with a less than 25 nm radius typically have a fairly low suspension turbidity. As the particle size increases, the turbidity increases to a maximum in the range of approximately 700 nm radius [147,148,149]. As the refractive index contrast between the particles and the surrounding medium increases, the turbidity or opacity increases. If the concentration of colloidal particles increases at this point, the opacity is further enhanced. In this regard, the size, concentration, and refractive index of the colloidal particles can be controlled to adjust the overall appearance of the food system for different food systems, such as clarified fruit juice and turbid milk.

## 4. Application of Food Colloids in Delivery Systems

### 4.1. In Vitro Instability

Some BACs, including probiotics, are involved in the regulation of organismal health and the prevention and treatment of chronic diseases. Still, they generally suffer from poor solubility, easy degradation, and low bioavailability (Table 3) [150]. These problems can be effectively solved by constructing a BAC delivery system that is compatible with the food system and does not affect the sensory properties of the food [38].

#### 4.1.1. Physical Instability

The instability of BACs is partly related to their physical properties. For example, polyvalent mineral ions (e.g., calcium, copper, or iron) can promote the precipitation of some proteins and the gelation of some polysaccharides, for which food colloids can be used to isolate unstable actives from unfavorable environmental factors [151]. In some water-based foods, the addition of fat-soluble actives may cause delamination. In this regard, we can use encapsulating materials to modify the surface properties of the particles. For example, the solubility of fat-soluble vitamin D was significantly increased by molecular self-assembly and co-assembly embedded in β-lactoglobulin [152]; the aqueous solubility of linoleic acid embedded using β-lactoglobulin nanocapsules was increased from 12% to 45% [153]; and the aqueous solubility of coenzyme CoQ10 embedded using albumin nanoparticles was significantly improved [154].

#### 4.1.2. Chemical Instability

Some BACs are also unstable under certain chemical conditions, such as changes in hydrogen ions, transition metals, oxidizing agents, reducing agents, and light. Encapsulation can effectively protect these BACs from any component that promotes chemical degradation or unfavorable environmental factors. For example, a major challenge for many vitamin applications is poor solubility and sensitivity to oxidation, and their dispersion and stability can be significantly improved using encapsulation. Researchers have successfully embedded vitamin E in modified tapioca starch nanocapsules, significantly improving their thermal stability and maintaining good solubility [155]; Vitamin D is hydrophobically bound to nanomicelles formed by the self-assembly of casein-hydrolyzed amphiphilic peptides, which significantly improves its thermal stability [156]; Vitamin C is susceptible to light, oxygen, and heat, whereas Vitamin C embedded in an inorganic SiO_2_ carrier retains 95% of the Vitamin C after one month of storage [157]. Additionally, polyunsaturated fatty acids, such as DHA and EPA, are susceptible to oxidation, and the embedding technology can prevent their oxidative deterioration and mask their fishy odor. Nanocapsules formed from pectin and β-lactoglobulin can protect DHA from degradation under processing and storage conditions, reducing its loss from 80% to 5–10% [158].

**Table 3:** Summary of functional properties and destructive factors of BACs.

Form	BACs	Functional Properties	External Influencing Factors	Body Absorption Disorders	Reference
Polyphenol	Curcumin	Used in anti-inflammatory and antioxidant tumor therapy against cardiovascular diseases, nervous disorders, inflammatory diseases, Alzheimer’s disease	Heat, light, iron	Poor solubility	[159]
	Resveratrol	Effectively scavenging free radicals; regulating the expression of antioxidant enzymes; anti-inflammatory, anti-aging, anti-diabetic, and cardioprotective effects	Thermal, light-induced isomerization, autoxidation	Low solubility/permeability, rapid metabolism	[160]
	Quercetin	Antioxidant, anti-inflammatory, anti-cancer	Heat, alkali	Low solubility and bioavailability	[161]
	Anthocyanin	Antioxidant, anti-inflammatory, anti-cancer, and chronic disease improvement (eyes, mouth, skin, colon, etc.)	pH, heat, oxygen, light, metal ions	Low solubility and bioavailability; strong acidic environment in the GI tract	[162]
Bioactive peptide	Carnosine	Antioxidant, pH buffering, chelation; anti-aging and anti-advanced glycosylation end products (AGEs)	-	Digestive tract degradation leads to low bioavailability and targeted delivery requirements	[163,164,165]
Bioactive polysaccharide	Lentinan	Antioxidant; combats inflammatory diseases and cancer	Heat, light, humidity	Low bioavailability, targeted delivery requirements	[166]
	*Ganoderma lucidum* polysaccharides	Anti-tumor, anti-inflammatory, immunomodulatory, and anti-aging agent effects; improvement in intestinal barrier function	Heat, light, moisture	Low solubility, low bioavailability, tumor environment (oxidative and acidic conditions)	[167]
Vitamin	Vitamin C	Increased collagen biosynthesis; reduced melanin synthesis; anti-aging; important for metabolic activity as a coenzyme or prosthesis	Heat, light, moisture	Low solubility, environmental degradation in the digestive tract	[168]
	Vitamin E	Anti-cancer, anti-aging	Heat, light, humidity, alkaline	Low solubility, environmental degradation in the digestive tract	[169]
Carotenoid	Lutein	Protects the retina; protects against UV-induced peroxidation; reduces lipofuscin formation and related oxidative stress-induced damage	Heat, light, oxygen	Low solubility, low bioavailability, gastrointestinal degradation	[170]
	β-carotene	Improves vision; strengthens immune system and cognitive function; prevents coronary heart disease and cancer	Heat, light, oxygen	Low solubility, low bioavailability, gastrointestinal degradation	[171]
Other	Isoflavone	Endocrine regulation, cardiovascular protection, antioxidant, anti-inflammatory, maintenance of bone health	Heat	Digestive tract degradation, low drug permeability	[172]
	β-sitosterol	Anti-inflammatory, anti-aging, anti-tumor, lipid-lowering effect and immunomodulation, positive effects on cardiovascular and cerebrovascular diseases	Temperature (volatile)	Low bioavailability due to low solubility and structural instability	[173]

### 4.2. Efficient Absorption In Vivo

After improving BACs’ aqueous solubility and stability in vitro, delivery vehicles face many obstacles to efficient absorption in vivo. Firstly, the human body environment is generally hydrophilic, so the poor aqueous solubility of hydrophobic compounds can greatly impede their dissolution in aqueous media and absorption under human gastrointestinal conditions (Table 3). Moreover, the human oral and gastrointestinal tract contains a large number of enzymes; many BACs in the process of transport may occur during chemical or biochemical degradation; e.g., flavonoids and hydroxycinnamic acids degrade by 84% and 80%, respectively, under in vitro gastrointestinal conditions, and vitamin C degrades by 91% under entero-digestive conditions [174]. Additionally, many peptides, proteins, and probiotics may be denatured or hydrolyzed in the stomach due to gastric juice’s high acidity, high enzyme activity, and high-bile-salt environment [89]. Only after this series of processes, the remaining BACs are further absorbed and utilized through active endocytosis in the small intestine [89]. However, the intestinal mucus barrier in the upper part of the small intestinal epithelium, which is a protective barrier in nature, will hinder the diffusion of certain BACs. In this regard, the design of smarter, more stable environmentally responsive release carriers is necessary: to solubilize, stabilize, and protect these BACs, targeting to overcome the gastrointestinal tract’s low pH, ionic strength, enzyme, and mucus barriers [175]. In addition, even when some small molecules reach the intestinal cell layer, they still have difficulty entering the body’s circulation through cellular tight junctions. The cellular uptake mechanism is limited by multi-drug resistance (multi-drug resistance), and the presence of the glycoprotein efflux pump p-gp on the intestinal epithelial cells, which pumps the absorbed substances back into the lumen, further reducing their intestinal absorption rate. In this regard, it has been shown that hydrophilic and charge-neutral particles can easily cross the mucus [176]. Additionally, nanocapsules can directly enter small intestinal epithelial cells through the active pathway and reversibly open tight junctions between epithelial cells, enhancing the delivery of BACs through the paracellular pathway [177]. Preparation of target ligand-conjugated nanocapsules can also promote absorption through their interaction with specific receptors on the surface of small intestinal epithelial cells [178]. It can be seen that multifunctional nanocarriers prepared based on hydrogel or self-assembly technology in response to the physiological conditions of the gastrointestinal tract (pH, enzymes, and intestinal mucus barrier) are more promising research directions [179].

Food delivery systems are now also becoming increasingly functional, not only responding to the physicochemical conditions of the GI tract and overcoming problems of biorecognition and intestinal mucus barrier diffusion but also controlling the release characteristics of the active substance through the design of the carrier. For example, gut-targeted carriers can release their encapsulated substances through degradation by intestinal microbial enzymes. In addition, some mucosal adhesion carriers can delay the release of entrapped substances and control their local concentrations near the surface of epithelial cells by interacting with mucus [180]. In conclusion, the targeted controlled-release delivery carrier system will be developed to become more and more intelligent, not only to become the emerging interdisciplinary cross-frontier research direction in the field of food, but also to provide the theoretical basis and technical support for the development of precision nutritional food.

Nowadays, in vitro simulated gastrointestinal tract modeling (e.g., the simulated gastrointestinal fluid model, the SHIME model, the TIM model, and the HGS model have provided an efficient alternative method for studying BACs’ digestion and absorption process without the need for animal or human experiments [89,181]. The model is low-cost, timesaving, and highly reproducible, and it has been widely used in the study of functional foods and drugs. In the future, the carrier–mucus interactions, carrier cellular uptake mechanism, targeted delivery mechanism, carrier metabolism safety, and the effects on intestinal flora should be investigated in depth based on these techniques. In addition, using wall materials with prebiotic properties to construct gut-responsive release carriers to deliver highly active probiotics has a broad research and application prospect.

### 4.3. Flavor Regulation

**Figure 4:**
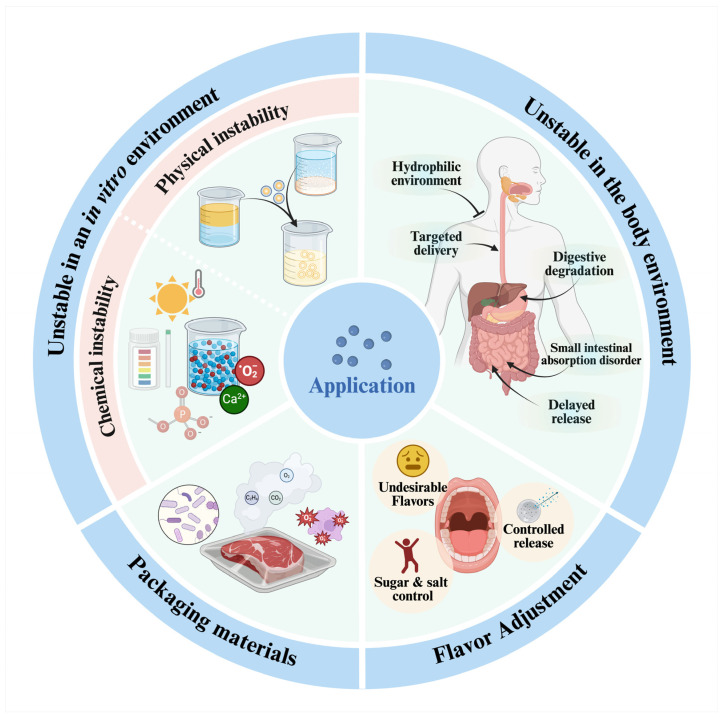
Application of food colloids in delivery systems.

Nowadays, to promote human health, there is a growing interest in functional foods that are low in fat, sugar, and salt or rich in BACs. At the same time, consumers want foods with desirable organoleptic properties (texture and flavor). Maintaining the health properties of foods without sacrificing their flavor is a great challenge, and the release of flavor substances is not only influenced by the composition of the food, but also by the structure of the food [182]. Researchers have been working on designing release systems for flavor compounds that have desirable release properties as they undergo processing, storage, and oral chewing (Figure 4). As mentioned earlier, the release characteristics of flavor compounds from food delivery vehicles are usually influenced by the nature of the colloid. Stefania Karaiskou et al. observed slower flavor release in emulsion systems with higher viscosity containing pectin or gum arabic [183]. Compared to gelatin, the effects of pectin and starch on flavor release were relatively weak, probably due to their less-compact gel network structure [184]. pH adjustments also affected the pK values of flavor compounds, which altered flavor distribution and release [182]. For example, in egg yolk or starch sodium octenylsuccinate-stabilized emulsions, an increase in pH from 3.0 to 9.0 resulted in enhanced retention of diacetyl, which was mainly due to enhanced electrostatic attraction or hydrogen bonding between diacetyl and the stabilizer under alkaline conditions [185]. In particular, some ingredients with specific bioactive effects may have unsatisfactory flavors that affect the overall taste of some food products. For example, some water-soluble peptides, proteins, and minerals have a bitter or astringent taste; fish oils have an undesirable fishy-like taste; etc. [124] For these components, encapsulating them in some sort of colloidal delivery system can effectively reduce their release in the oral cavity and their interaction with taste receptors in the mouth or mucins in the saliva, thereby inhibiting the production of undesirable flavors [177]. It is also important to ensure that such substances break down after swallowing so that the BACs are released at the target site. For example, Zekun Li et al. were able to encapsulate fat-soluble capsaicin with its hydrophobic core using whey protein nanomembranes, which not only improved its water solubility but also masked its pungent irritation, and in addition, the nanomembranes exhibited intestinal-responsive release behavior [186].

### 4.4. Packaging Materials

Food packaging plays a crucial role in food quality, safety, and extended shelf life. Over the past few years, many innovations and advances in packaging technology have emerged, including intelligent or smart packaging (i.e., time–temperature indicators, gas indicators, and radiofrequency identification), and active packaging (i.e., oxygen scavengers, moisture absorbers, and antimicrobials), which have been instrumental in improving food quality, safety, and shelf life (Figure 4) [187]. Among them, active packaging also utilizes the principle of colloidal delivery. According to the mechanism of action of different active packaging, they can be classified into “release systems” (e.g., enzymes, antimicrobial agents, antioxidants, and carbon dioxide or ethanol releasers), “absorption systems” (e.g., oxygen, carbon dioxide or ethylene scavengers, and moisture or aroma absorbers) and “non-migratory systems” (e.g., antimicrobial nanoparticles) [188]. They use packaging materials as carriers in which active compounds are incorporated, retained, and released appropriately to protect or enhance food quality. For example, the incorporation of green tea extract into inorganic packaging for meat resulted in an average three-day extension of the shelf life of fresh meat [189]. Chitosan derivatives have been shown to have excellent antimicrobial activity and silver nanoparticles of cellulose [190,191], and chitosan and fucoidan biopolymers have been prepared for antimicrobial applications [192]. Other interesting polysaccharides used for antimicrobial packaging are alginate and carrageenan [193]. The loading, delay, and release of active compounds from encapsulated compositions are usually influenced by external factors, such as removing moisture, heat or barrier layers, or intermolecular forces between the packaging material and the food component. However, it is worth mentioning that most current studies have been conducted using liquid simulants under sufficient agitation, and it is well known that the release of active compounds is much slower in real food than in liquid food simulants [194,195]. Therefore, the results obtained in food simulants are useful for developing kinetic models to predict whether the release of active compounds in real food is effective, and how it can be improved is a future need for further consideration.

## 5. Conclusions

In recent times, significant progress has been made in the study of colloidal delivery systems for BACs; particularly, the food colloid-based delivery of BACs has attracted increasing attention because of its safety, efficiency, and controllability. This review systematically introduces several representative food colloid delivery carrier structures, including liposomes, Pickering emulsions, hydrogels, nanomicelles, and cyclodextrins, summarizes the advantages and limitations as well as the static-to-dynamic physical properties of these colloidal delivery carriers, and pays special attention to their possible impact on the physical properties of the product matrices upon application. In addition, this review discusses the possible application scenarios and future perspectives of food colloidal delivery carriers with respect to the problems and challenges encountered in the application of BACs. The complexity of different colloidal delivery systems in the digestive process makes it necessary to further explore the relationship between changes in colloidal structure and the release mechanism of BACs and to summarize and establish relevant mathematical models to help form a systematic study. Compared to the research process of drug delivery systems, most of today’s studies on food colloid delivery systems focus on the design of in vitro stability and solubility of BACs, while studies about in vivo release control are still lacking. Therefore, future research needs to build on the established mathematical models to explore or artificially synthesize more malleable and controllable food-derived polymers to develop commercially viable colloidal delivery systems that can release payloads at specific locations in the human gastrointestinal tract, such as the oral cavity, stomach, small intestine, or colon, which can help to personalize personalized nutritional foods for different health states in the future. In particular, unlike traditional drug delivery, future developments will also require BACs to be stably delivered in complex food matrices, for which the stability of colloidal particles in product matrices and the impact on product quality will also need to be considered. In addition, in order to facilitate the implementation of the research technology into practical applications, the possible food processing conditions that may be employed should also be considered to test the colloidal stability. Overall, significant challenges remain in preparing colloidal delivery systems that are economically viable, are robust enough to be incorporated into food products, do not alter desirable product properties, and are effective in retaining, protecting, and releasing specific actives.

## Figures and Tables

**Table 1:** Application of food colloidal delivery systems with different structures.

Structure	Delivered Substances	Colloidal Materials	Processing Technology	Function	References
Liposome	Curcumin	Phosphatidylcholine, cholesterol	Detergent removal method	Improved stability, solubility in aqueous medium	[7]
	Shrimp oil	Soy lecithin (L-α-Phosphatidylcholine)	Ultrasonication and microfluidization method	Prevents oxidation of oil during storage and masks the undesirable fishy odor	[7,22]
	Lycopene	Soy lecithin, cholesterol, β-CD	Thin-film hydration method	Enhances in vivo activity; controls release	[23]
	Green tea polyphenols	-	Microencapsulation	Improve the stability of polyphenols in food processing	[6]
Pickering emulsion	Carotenoids (β-carotene)	Wheat gluten nanoparticles	-	Improve stability to chemical degradation during storage and enhances its bioaccessibility in a simulated gastrointestinal tract	[24]
	Water-insoluble phytosterols	Phytosterols, whey protein concentrate	Anti-solvent method	Improved stability, bioavailability	[25]
	Oleuropein	Pectin, whey protein	Sonication	Encapsulation efficiency of 91% is achieved	[26]
	Betanin, curcumin	Sugar beet pectin, bovine serum albumin nanoparticles	Genipin cross-linking strategy	Improve their thermal stability and bioavailability	[27]
	Anthocyanin	Octenylsuccinate quinoa starch	-	Improves storage stability	[28]
	Epigallocatechin-3-gallate, quercetin	Gelatin, gliadin nanoparticles	Two-step procedure	Improve EGCG chemical stability and quercetin solubility under simulated gastrointestinal conditions	[29]
Hydrogel	*Lactobacillus plantarum*, *Lactobacillus salivarius*	Peach gum polysaccharide and *Auricularia polytricha* β-glucans	Inverse emulsion cross-linking method	Potential application as intestinal-targeted delivery systems	[30]
	Green tea polyphenols	Salecan and *N*,*N*,*N*-trimethyl chitosan	Self-assembly	Efficiently encapsulated into PEC hydrogels and liberated in a sustained pattern	[31]
Self-assembled microcapsules based on electrostatic interactions	Red pigment from paprika	Casein, carrageenan	Ultrasonic Maillard dry treatment	Enhances thermal, enteric, and storage stability for free release and emulsification	[30]
	Ovalbumin	RGD peptide-grafted carboxymethyl starch, cationic quaternary ammonium starch	-	Improves intestinal stability and delivery transportation efficiency	[32]
Self-assembled nanomicelle	Curcumin	Lactoferrin hydrolysate	-	Improves thermal, dilution, and storage stability as well as improved in vivo conversion and bioavailability	[33]
	Curcumin	Beta-casein	-	The antioxidant activity of curcumin encapsulated in B-CN is higher than that of both free B-CN and curcumin	[34]
Cyclodextrin	Quercetin	SBE-β-CD	Complexation in water	Significantly increases the solubility and bioavailability of quercetin	[35]
	*Trans*-polydatin	β-CD	Complexation in water/ethanol solution and freeze-dried	Increases solubility, thermal stability, photostability, and antioxidant activities of trans-polydatin after forming CICs.	[36]
	Blackberry anthocyanins	β-CD	Complexation in water and freeze-dried	Enhance thermal stability and bioaccessibility of blackberry anthocyanins after forming CICs	[37]

**Table 2:** Comparison of physical properties of food colloidal delivery carriers with different structures.

Carrier Classification	Size	Charged Characteristics	Binding Characteristics	Physical Stability	Release Behavior	References
Liposome	20 nm to a few microns	Surface charging properties are mainly dependent on the lipid composition contained (categorized as cationic/negative/neutral liposomes)	Adhesive interactions and chemical bonding between phospholipid molecules	Physical instability (thermodynamic instability), chemical instability (susceptibility to oxidation and hydrolysis), and biological instability	Mainly concerned with structural changes in liposome membranes and kinetic processes of drug release	[48]
Pickering emulsion	Micron level (depending on the process)	-	Capillary force and adsorption of solid particles at the interface	Surface wettability of solid particles, concentration, electrolytes in the aqueous phase, pH, and the volume ratio of the oil–water phase affect the adsorption behavior of solid particles at the oil–water interface	Stabilization of solid particles at the oil–water interface to control emulsions	[57]
Hydrogel	Size of hydrogel is a flexible and variable concept; there are large-size hydrogels at the macroscopic scale and nanoscale hydrogels at the microscopic scale	The situation is complex and is usually related to the charged groups of the polymer chain, surface ion adsorption, pH, and ion concentration in the environment	It can be formed by chemical cross-linking (covalent bonding) or physical cross-linking (non-covalent bonding) of macromolecules. Chemical cross-linking has been widely used in food macromolecule modification, food packaging, microsphere preparation, and other applications.	Dehydration-induced hardening and softening phenomena, polymer aggregation, and stabilizer selection and use	Primarily concerned with their physical and chemical properties and how they affect drug release	[74]
Complex coacervates	Usually about 10–200 nm	Mainly in the nature and distribution of its surface charge	Electrostatic force	The pH and ionic concentration of the surrounding medium, temperature, etc., affect its stability	Usually depends on disintegration mediated by external conditions	[83]
Layer-by-layer	Nanometer scale	Mainly in the nature and distribution of its surface charge	Weak interactions (e.g., electrostatic interactions, hydrogen bonding, coordination bonding, etc.)	The pH and ionic concentration of the surrounding medium, temperature, etc., affect its stability	Precise control and release of substances can be achieved by controlling the interactions between layers	[93]
Self-assembled nanomicelles	Between a few nanometers and a few tens of nanometers	Depends mainly on the type of surfactant it contains	Interactions between non-covalently bonded molecules such as hydrogen bonding, hydrophobic interactions, electrostatic interactions, etc.	Mainly concerned with their dynamic properties and preparation methods	May be related to the dissociation of micelles, which may occur when external environmental conditions change	[33]
Cyclodextrin	Depends on its specific type	Electrically neutral	-	This is mainly related to its structural characteristics and some unfavorable factors in the application environment	Decomposition of the inclusion by adjusting its structure and properties	[106]

## Data Availability

No new data were created in this study. Data sharing is not applicable to this article.
